# Neural Correlates of Infant Face Processing and Later Emerging Autism Symptoms in Fragile X Syndrome

**DOI:** 10.3389/fpsyt.2021.716642

**Published:** 2021-11-24

**Authors:** Maggie W. Guy, John E. Richards, Abigail L. Hogan, Jane E. Roberts

**Affiliations:** ^1^Department of Psychology, Loyola University Chicago, Chicago, IL, United States; ^2^Department of Communication Sciences and Disorders, Arnold School of Public Health, University of South Carolina, Columbia, SC, United States

**Keywords:** event-related potentials, fragile X syndrome, autism spectrum disorder, infant, child

## Abstract

Fragile X syndrome (FXS) is the leading known genetic cause of autism spectrum disorder (ASD) with 60–74% of males with FXS meeting diagnostic criteria for ASD. Infants with FXS have demonstrated atypical neural responses during face processing that are unique from both typically developing, low-risk infants and infants at high familial risk for ASD (i.e., infants siblings of children with ASD). In the current study, event-related potential (ERP) responses during face processing measured at 12 months of age were examined in relation to ASD symptoms measured at ~48 months of age in participants with FXS, as well as siblings of children with ASD and low-risk control participants. Results revealed that greater amplitude N290 responses in infancy were associated with more severe ASD symptoms in childhood in FXS and in siblings of children with ASD. This pattern of results was not observed for low-risk control participants. Reduced Nc amplitude was associated with more severe ASD symptoms in participants with FXS but was not observed in the other groups. This is the first study to examine ASD symptoms in childhood in relation to infant ERP responses in FXS. Results indicate that infant ERP responses may be predictive of later symptoms of ASD in FXS and the presence of both common and unique pathways to ASD in etiologically-distinct high-risk groups is supported (i.e., syndromic risk vs. familial risk).

## Introduction

Fragile X syndrome (FXS) is a single-gene disorder that results from a CGG repeat expansion mutation on the X chromosome affecting approximately one in 3,700–8,900 males (1–4) and one in 11,100 females (4). FXS possesses a high level of comorbidity with autism spectrum disorder (ASD) and is the most common single-gene cause of ASD, evidenced by 60–74% of individuals with FXS meeting diagnostic criteria for ASD (5–9). This is much higher than the rate of 1.9%, which is observed in the general population (10). Research has increasingly focused on early detection of ASD in FXS with evidence suggesting that 61% of preschool children meet diagnostic criteria with a high degree of diagnostic certainty (9). Likewise, behavioral risk markers for ASD are present in infants with FXS by 12 months of age (11, 12) and are predictive of later ASD diagnoses (9). In addition, specific neural responses [i.e., event-related potentials (ERPs)] related to ASD are also atypical in infants with FXS at 12 months of age (13). However, the relationship between neural responses in infancy and ASD symptom severity in early childhood have not been reported as we do in the current study. Research on the early development of ASD symptoms in infants and children with FXS provides insight into multiple developmental pathways to ASD, which may improve identification of reliable risk markers in infancy and facilitate earlier diagnosis and intervention.

To date, nearly all of the research examining early risk markers of ASD has been conducted on infant siblings of children diagnosed with ASD (henceforth referred to as ASIBs), who are at elevated risk for ASD because of the significant heritability of ASD. Approximately 20% of ASIBs will be diagnosed with ASD themselves (14–16), and another 20–40% will exhibit other developmental differences (14). A review of literature on the development of ASD in ASIBs found that the first behavioral signs of ASD typically emerged between 6 and 18 months of age and were most frequently associated with atypical patterns of social attention (16). Other behavioral studies have shown that infants at high risk for ASD demonstrate decreased attention to social stimuli, including faces [e.g., (17, 18)]. In one study, this was reported in infants as young as 1 week old (19). Still, there are additional studies that do not report early atypical social attention in high risk infants [e.g., (20, 21)].

Recent work focused on understanding the early signs of ASD in infants with FXS suggests that those infants with FXS that are later diagnosed with ASD demonstrate early social-communicative deficits similar to those observed in ASIBs later diagnosed with ASD (9, 11). In a series of case studies, Hogan and colleagues (11) followed eight infants with FXS longitudinally from 9 to 24 months of age. They found that the four infants with FXS that were later diagnosed with ASD demonstrated impairments in social communication, including reduced social interest, social smiling, and babbling, which were not consistently displayed in the four infants that were not later diagnosed with ASD. Additional work has shown that atypical eye contact (9) and social avoidance (22) in infancy are also associated with greater severity of ASD symptoms in young children with FXS. Although not necessarily social in nature, atypical patterns of visual attention (23) and physiological arousal (9, 24) have also been related to the presentation of symptoms of ASD in infants with FXS.

Despite significant progress in understanding how behavioral symptoms of ASD emerge and change across early development (16), much remains to be learned about heterogeneity in the early development of ASD and the presentation of reliable risk markers in the first year of life. As subsets of both infants with FXS and ASIBs show behavioral ASD symptoms that emerge in infancy and are predictive of later diagnoses of ASD, examining early neural risk markers in these groups from a cross-syndrome approach may lead to an increased understanding of each group individually, as well as inform understanding of heterogeneous pathways to ASD (25). Recent work indicates that atypical infant brain responses are early-appearing and reliable indicators of ASD risk in ASIBs (26). Specifically, atypical patterns of neural activation during face processing have been reported in studies of infants with FXS (13) and ASIBs [e.g., (13, 27–30)]. Benefits of an ERP approach to investigating risk markers for ASD are seen in their ability to detect unique patterns of brain activity, which may emerge prior to behaviors associated with ASD and may be evident in an infant sample that is inherently limited by a restricted range of behaviors. Additionally, ERP measures may provide a more objective measure of risk than assessment of behavior. In the current study, we investigated the possibility that neural responses to social (i.e., face) and non-social (i.e., toy) stimuli in infancy, measured through ERPs, may be associated with later-emerging symptoms of ASD in infants with FXS contrasted against etiologically-distinct high-risk infants (ASIBs) and low-risk infants.

In the first months of life, face processing is believed to occur within a subcortical pathway that is recruited to a lesser extent with age as cortical pathways become specialized for face processing (31, 32). If early visual attention to social stimuli is similar across high- and low-risk infants, it may be because they rely on the same, intact subcortical pathway (31). From this perspective, deficits are expected to emerge near the end of the first year of life, as cortical pathways become established, and show atypical function associated with emerging ASD (31, 33). Further evidence that the timing of cortical pathways influence social attention near the end of the first year of life comes from behavioral work recently described by Ozonoff and Iosif (34), indicating that a regression in social attention (e.g., eye contact) from 6 to 12 months of age was present in 86% of ASIBs that went on to receive a positive diagnosis for ASD. They report that group differences in social attention are rarely seen before 9 months of age, but seem to emerge around 12 months and then increase in magnitude with age. Taking this evidence into account, it has been posited that deficits in the function of the “social brain” may become evident earlier in development at the neural level than at the behavioral level. ERPs show excellent sensitivity to neural timing and patterns of stimulus responses and have the potential to show high sensitivity for identifying atypical responses to stimuli, such as faces, in infancy, before behavioral symptoms of ASD manifest. As such, the use of ERPs in research with high-risk infants may allow for the identification of an early, reliable marker associated with the later emergence of ASD.

In typically developing infants, the N290 and P400 ERP components have been identified as possible precursors to the N170 ERP component, which is associated with face specialization in adults [e.g., (29)]. The N290 peaks ~290 ms after stimulus onset and is most similar to the N170, as they are both negative peaks observed at lateral posterior electrode sites [e.g., (35, 36)]. Like the N170, the N290 is greater in amplitude in response to faces than other stimuli (35, 37). The P400 is a positive amplitude ERP component that peaks at ~400 ms after stimulus onset over occipital scalp sites (35, 36, 38). The role of the P400 in social information processing in infancy is less well understood. For example, some studies have reported shorter latency to faces than other stimuli (30, 37, 39), others have reported greater amplitude to non-face than face stimuli (36), and others have found no significant effects based on stimulus category (13, 35).

The N290 and P400 have also been investigated in infant ASIBs, although the effect of risk on neural correlates of face processing is not straightforward. Some studies have found greater amplitude N290 response to faces than other classes of stimuli in ASIBs (13, 30). Luyster and colleagues (29) conducted a large-scale longitudinal study of ERP components in 61 ASIBs and 70 low-risk control (LRC) infants from 6 to 36 months of age. Their results indicated similar developmental trajectories of the N290 and P400 across groups, and included only marginally significant group differences in N290 responses to the infant's mother's face vs. a stranger's face. They found that LRC infants demonstrated greater differentiation of these stimulus categories than ASIBs. Additionally, there was a marginally significant interaction of participant group and stimulus category on P400 amplitude. Nine ASIB participants later received an ASD diagnosis, however, the authors reported that inclusion or exclusion of participants based on ASD diagnosis did not significantly impact the results.

An additional ERP component that is of great interest in infant research is the Negative central (Nc), which occurs ~350–750 ms after stimulus onset at midline frontal and central electrodes (40). The Nc is not directly associated with social processing but is indicative of attentional engagement and is observed in response to a wide range of visual stimuli. Nc amplitude is typically greater in response to novel or salient stimuli than familiar stimuli (39, 41–44). Studies measuring infant heart rate responses have also found that Nc amplitude is greater during heart rate-defined periods of sustained attention (36, 45). The Nc is of interest to the current investigation, as it may provide insight into the presence of atypical attentional allocation in infancy, which would be expected to reflect more general processing deficits, less closely associated with social information processing specifically.

Guy and colleagues (13) conducted the first investigation of neural correlates of face processing in multiple groups of infants at high risk for ASD, including infants with FXS and ASIBs. ERPs were measured in response to familiar and novel faces and toys. Across participant groups, a greater amplitude N290 was observed to faces than toys. Differences in N290 amplitude to faces and toys were most pronounced in infants with FXS and smallest in ASIBs. Additionally, visual examination of the data revealed that infants with FXS showed an enhanced N290 response relative to the other two participant groups. This was reflected in a significant group by stimulus familiarity interaction. Infants with FXS showed greater N290 amplitude to familiar stimuli than novel stimuli, while other participants did not discriminate stimuli based on familiarity at the N290. Furthermore, responses to familiar stimuli in infants with FXS were greater than ASIBs' and LRC infants' responses to familiar and novel stimuli. No significant differences were observed for the P400 across group or stimulus type. At the Nc ERP component, ASIBs demonstrated a more muted response than infants with FXS and LRC infants. Although Nc amplitude did not differ across face and toy stimuli, there was an effect of stimulus familiarity on Nc responses. LRCs showed a greater Nc response to novel (i.e., a stranger's face, a novel toy) than familiar stimuli (i.e., their mother's face, a favorite toy). Infants with FXS showed a greater Nc response to familiar stimuli as opposed to novel stimuli, which has been observed in some research conducted with younger (i.e., 6-month-old) infants with typical development (39, 41, 43). Interestingly, ASIBs did not differentiate stimuli based on familiarity.

These results indicate that while both infants with FXS and ASIBs are at an increased risk of developing ASD, differing patterns of neural responses to social and non-social stimuli are observed across these two etiologically-distinct high-risk groups. Not only do their ERP responses differentiate them from low-risk control infants, but also from each other. What remains to be known is whether group differences in N290 amplitude responses in infants with FXS and ASIBs may be associated specifically with emerging symptoms of ASD. The enhanced N290 in infants with FXS could reflect a hyperactive or hypervigilant neural response to social stimuli that could be related to later social anxiety, which is highly prevalent in FXS [e.g., (46)]. Furthermore, a more muted response in ASIBs may reflect a reduced, hypoactive response indicating reduced social interest and salience, as has been observed in individuals with ASD [for review see (47)].

The objective of the current study was to determine how the neural correlates of faces processing in infancy relate to ASD symptoms later in childhood in children with FXS compared with another group at high risk for ASD (i.e., ASIBs) and LRC children. In the current study, we utilized previously collected ERP data from high-risk infants and LRCs (13) to examine the relations between infant ERP responses during a face processing task and ASD symptom severity in early childhood. Infant ERP responses were measured at 12 months of age and clinical assessment for ASD was conducted later in childhood, at ~48 months of age. In an approach similar to that used by Elsabbagh and colleagues (48), relations between N290, P400, and Nc amplitude and continuous scores of ASD symptom severity were investigated. Severity scores were used to quantify overall ASD symptoms, social symptoms, and restricted and repetitive behavior symptoms. We hypothesized that N290 responses to faces would be associated with overall symptom scores, as well as social scores, but that the pattern of relations would vary across high-risk groups. Specifically, we expected that enhanced N290 amplitude in infants with FXS would be related to higher ASD symptom scores, while muted N290 amplitude in ASIBs would be associated with higher ASD symptom scores. As the N290 is uniquely sensitive to face stimuli, we did not believe that N290 responses would be closely linked to restricted and repetitive behavior scores. Additionally, based on the hypothesized role of the P400 in social information processing, we predicted that the P400 may be associated with overall or social affect symptom scores, but that it would be less closely correlated with restricted and repetitive behavior scores. However, due to the observation of similar P400 responses across infants with FXS, ASIBs, and LRCs in previous research (13), we expected that the P400 would be less likely to be significantly related to ASD symptoms than the N290. Differences were previously observed across groups in Nc responses, but based on the Nc's responsiveness to a wide range of visual stimuli, we believed that the Nc may be more broadly associated with overall ASD scores and not linked with a specific domain of ASD symptoms. For example, relations between Nc responses and symptoms of ASD were not expected to be face specific, and to have connections to symptom severity scores across the three different scales. We examined relations across face and toy and familiar and novel stimuli, based on significant effects observed in this group in infancy (13).

## Methods

### Participants

Fifty 12-month-old infants were included in the study, including 14 infants with FXS (seven males), 18 ASIBs (15 males), and 18 typically developing low-risk control (LRC) infants (14 males). All participants were retained from our previous research study (13). An additional infant with FXS, three ASIBs, and three LRC infants were tested in the original Guy et al. (13) study, but were not retained in the current study due to lack of outcome data. Infants with FXS were identified through collaborations with researchers across the United States in addition to emails and postings through social media. ASIBs were recruited through the South Carolina Department of Disabilities and Special Needs; a letter was sent to families with a child with an ASD diagnosis, inviting participation from families with an infant sibling. LRC infants were recruited from the Columbia, SC area and were required to have no known developmental anomalies and no family history of ASD or related disorders (e.g., FXS). Participants were recruited without regard to race, ethnicity, socioeconomic status, and gender. However, participants were primarily Caucasian and of middle socioeconomic status. All infants participated with the informed, signed consent of their parents.

### Measures and Apparatus

#### EEG (Infant Timepoint)

EEG was recorded in 12-month-olds using the Electrical Geodesics, Inc. (EGI) high-density 128-channel EEG system. Participants were seated on a parent's lap during the recording. They were positioned about 55 cm from a 29″ LCD monitor (NEC Multisync XM29). A video camera was just above the monitor and used to record participant looking behavior. An experimenter judged infant fixation online and controlled stimulus presentation using EGI Net Station and E-Prime software in an adjacent room. Stimuli included photographs of female faces (i.e., the mother's face and a stranger's face) and infant toys (i.e., a picture of a toy belonging to the infant and a novel toy). Sesame Street video clips were used as attractors when children lost interest in the stimuli. All stimuli were presented on colorful, variegated backgrounds [see (13) for more details].

#### ASD Symptoms (Outcome Timepoint)

Participants were followed longitudinally as part of a larger study on development in high-risk infants, and ASD symptoms were assessed annually at outcome timepoints beginning at 24 months of age. For the current study, we targeted clinical data from their 36-month visit or later, as ASD symptoms and diagnoses assessed at this age appear to be stable (9, 49, 50). Data from the 24-month visit were used if no later data were available (*n* = 0 children with FXS, *n* = 2 ASIBs, *n* = 2 LRC children). Although assessment of ASD symptoms prior to 3 years of age are generally considered less reliable, recent research has provided promising evidence of symptom and diagnostic stability starting at 18 months (51–53). The mean age of outcome assessment was similar across groups (ASIB: *M* = 48.00 months, FXS: *M* = 47.14 months, LRC: *M* = 44.67 months).

The Autism Diagnostic Observation Schedule−2^nd^ Edition [ADOS-2; (54)] was used to measure ASD symptoms. Overall, Social Affect (SA), and Restricted and Repetitive Behavior (RRB) calibrated severity scores (CSS) were computed using established guidelines (55, 56). The CSS has been established as a stable continuous measure of ASD severity that is more valid than the overall ADOS raw score (57). Average calibrated severity scores are presented by group in Table 1.

**Table 1:** ADOS calibrated severity scores by participant group.

	**ASIB**	**FXS**	**LRC**
*n*	18	14	18
*n* male (%)	15 (83%)	7 (50%)	14 (78%)
Age in months at ADOS	48.00 (20.56)	47.14 (9.94)	44.67 (14.73)
Overall CSS (*SD*)	4.06 (3.00)	4.79 (2.46)	2.28 (1.78)
SA CSS (*SD*)	4.22 (2.73)	4.43 (2.77)	2.61 (1.94)
RB CSS (*SD*)	5.11 (3.31)	5.93 (3.17)	2.94 (2.62)

### Procedure

#### EEG

Participants were fitted with an EGI “hydrocel geodesic sensor net” (HGSN) that was selected based on their head circumference. Net application took 5–10 min, during which a second experimenter entertained the infant with toys. The experiment commenced once the infant was positioned in front of the monitor. An attractor stimulus was used to draw fixation toward the center of the screen and a button was used to indicate fixation and to begin stimulus presentation, which included brief stimulus presentations and visual paired comparison (VPC) trials. Brief stimulus presentations included a 100 ms blank screen baseline period, followed by a 500 ms stimulus presentation, and a variable inter-trial interval of 500–1500 ms. The VPC trials included side-by-side presentations of the two face stimuli or the two toy stimuli and lasted until 4 s of looking time was reached. The VPC and brief stimulus presentations were presented in random order in 10-trial blocks. If the infant looked away from the screen, the Sesame Street attractor stimulus was used to regain fixation toward the screen. Stimulus presentation continued until the infant became bored or fussy.

### Data Analysis

#### EEG Recording and Analysis

The EEG was recorded from 124 electrodes in the EGI HGSN, two additional electrodes measured electrooculogram (EOG), and two electrodes measured electrocardiogram (ECG). Recordings were referenced to the vertex online, recorded with 20K amplification at a 250 Hz sampling rate with bandpass filters set from 0.1 to 100 Hz and 100 kΩ impedance. Data processing was completed using the EEGLAB and ERPLAB Matlab toolboxes (58, 59). Following data collection, the vertex-referenced EEG was algebraically recomputed to the average reference. The EEG was filtered with a 0.5 Hz high-pass filter and ERP trials were segmented from 50 ms before stimulus onset through 1 s following onset. Recorded EEG was inspected for artifact (defined as a change in amplitude >100 μV), poor recordings, and blinks using the ERPLAB toolbox in Matlab and visual inspection. Trials were eliminated from further analyses if more than 10 channels were affected.

Clusters of virtual “10-10” electrodes were created from the mean of the EGI electrodes surrounding the traditional 10-10 electrode locations [see (36) Supplemental Information]. The N290 was examined at lateral posterior-inferior electrodes including Parietal Occipital (PO7: 59, 65, 66; PO8: 84, 90, 91; PO9: 64, 65, 68, 69; PO10: 89, 90, 94, 95), Parietal (P7: 51, 58, 59; P8: 91, 96, 97; P9: 57, 58, 63, 64; P10: 95, 96, 99, 100), and Temporal Parietal electrodes (TP7: 46, 50, 51; TP8: 97, 101, 102; TP9: 50, 56, 57; TP10: 100, 101, 107). The P400 was examined at medial posterior-inferior electrodes including Parietal Occipital (PO7–10), Occipital (Oz: 71, 75, 76; O1: 66, 70, 71; O2: 76, 83, 84), and Inion electrodes (Iz: 74, 75, 81, 82; I1: 69, 70, 73, 74; I2: 82, 83, 88, 89). The Nc was analyzed at frontal and central midline virtual electrodes (Fz: 5, 10, 11, 12, 16, 18; FCz: 5, 6, 7, 12, 106; Cz: 7, 31, 55, 80, 106). Additional information on the selection of time windows for ERP component analysis and computation of ERP amplitude can be found in our previous publications (13, 35, 60).

To better understand relations between early neural responses to visual social and non-social stimuli and later symptoms of ASD, we assessed ADOS-2 calibrated severity scores in relation to infant ERP responses. In extension of methods utilized in past research (13), amplitude of the N290, P400, and Nc ERP components based on stimulus type (2: faces, toys) and stimulus familiarity (2: familiar, novel) were examined in association with Overall, Social Affect (SA), and Restricted and Repetitive Behavior (RRB) CSS using ANCOVAs and regressions. A general linear models approach (i.e., “Proc GLM” of SAS) using nonorthogonal design was used. The statistical tests used error terms derived from the related interval effect analyses to control for inflation of test wise error rate. All significant tests are reported at *p* < 0.05 and effect sizes (eta squared) and 95% confidence intervals for effect sizes are reported to describe comparisons within significant effects.

## Results

### N290

**Figure 1:**
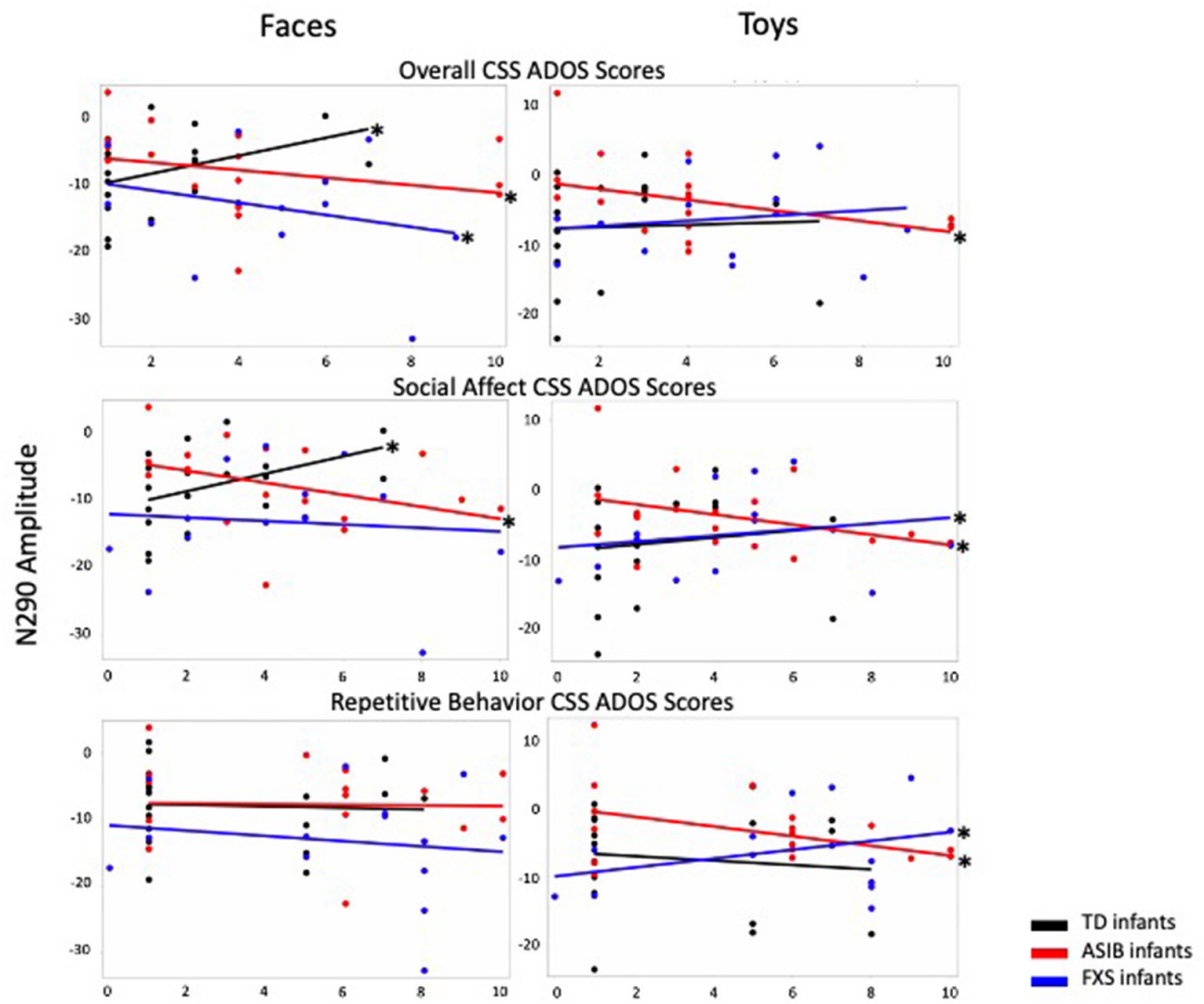
N290 amplitude to faces and toys across ASIBs, infants with FXS, and LRC infants is presented in relation to Overall, Social Affect, and Restricted and Repetitive Behavior calibrated severity scores. Average N290 amplitude is calculated across Parietal Occipital, Lateral Parietal, and Temporal Parietal electrodes.

Graphs presenting the relations between N290 amplitude to faces and toys across infants with FXS, ASIBs, and LRC infants and Overall, Social Affect, and Restricted and Repetitive Behavior CSS on the ADOS-2 are presented in Figure 1.

#### Overall CSS

There was a significant interaction between participant group, stimulus type, and Overall CSS on N290 amplitude, *F*_(2, 1188)_ = 10.05, *p* < 0.001, $n_{p}^{2}$ = 0.02, 95% CI [0.01, 0.03]. To better understand this interaction, we examined responses to faces and toys separately. In response to faces, there was a significant interaction of Overall CSS and group, *F*_(2, 594)_ = 15.65, *p* < 0.001, $n_{p}^{2}$ = 0.05, 95% CI [0.02, 0.09]. For infants with FXS and ASIBs, a more negative amplitude N290 in response to faces was associated with higher Overall CSS (infants with FXS: *F*_(1, 166)_ = 7.72, *p* = 0.006, $n_{p}^{2}$ = 0.04, 95% CI [0.00, 0.12]; ASIBs: *F*_(1, 214)_ = 9.66, *p* = 0.002, $n_{p}^{2}$ = 0.04, 95% CI [0.01, 0.11]). The opposite pattern of responses was seen for LRC infants, decreased (more positive) N290 amplitude in response to faces was related to higher Overall CSS, *F*_(1, 214)_ = 20.81, *p* < 0.001, $n_{p}^{2}$ = 0.09, 95% CI [0.03, 0.17]. In response to toys, there was a significant interaction of Overall CSS and group, *F*_(2, 594)_ = 9.08, *p* < 0.001, $n_{p}^{2}$ = 0.03, 95% CI [0.01, 0.06]. More negative N290 responses to toys were associated with higher Overall CSS for ASIBs, *F*_(1, 214)_ = 28.11, *p* < 0.001, $n_{p}^{2}$ = 0.12, 95% CI [0.05, 0.20]. N290 responses to toys were not significantly associated with Overall CSS for infants with FXS, *p* = 0.122, $n_{p}^{2}$ = 0.01, 95% CI [0.00, 0.07], or LRC infants, *p* = 0.598, $n_{p}^{2}$ = 0.001, 95% CI [0.00, 0.03].

**Figure 2:**
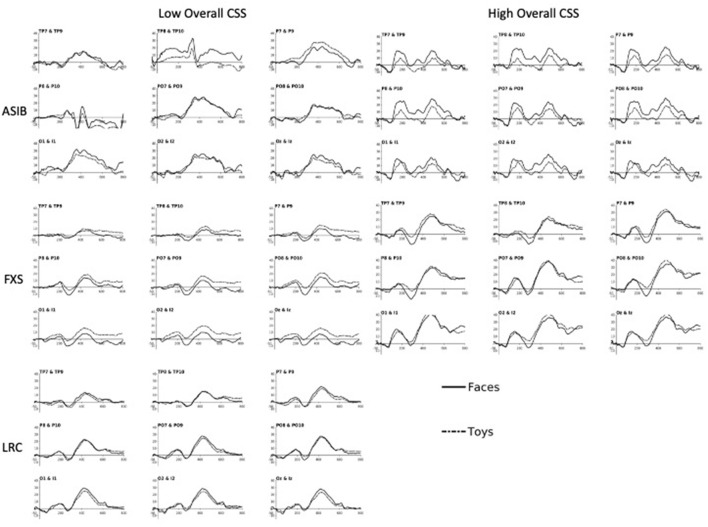
The N290 and P400 responses to faces and toys across subgroups of ASIB, FXS, and LRC participants. Plots labeled “Low Overall CSS” include participants with an Overall CSS of three or less on the ADOS-2. “High Overall CSS” includes participants with an Overall CSS of seven or higher on the ADOS-2. Participants scoring in the mid-range (i.e., scores of four to six) are not represented in these plots. There were not enough LRC participants to create “High Overall CSS” plots. Plots are presented at relevant “virtual 10-10 electrode” clusters, including Temporal Parietal (TP), Parietal (P), Parietal Occipital (PO), Occipital (O), and Inion (I) clusters [see (13, 36) for more details on calculation of 10-10 virtual electrodes].

Figure 2 presents ERP plots, which illustrate the effects described above using a small set of participants at each end of the range of possible scores. Plots labeled “Low Overall CSS” include participants with an Overall CSS of three or less on the ADOS-2. “High Overall CSS” includes participants with an Overall CSS of seven or higher on the ADOS-2. Participants scoring in the mid-range (i.e., CSS of four to six) are not represented in these plots. The N290 is evident as the negative deflection occurring ~300 ms after stimulus onset. The change in amplitude from the preceding peak of the P1 to the peak of the N290 is greater for ASIBs with high Overall CSS compared with ASIBs with low Overall CSS. N290 amplitude is also greater for participants with FXS and high Overall CSS, particularly in response to faces. LRC participants with low Overall CSS are included for comparison, however, there were too few high-scoring LRC participants for an average ERP plot in the “High Overall CSS” category. In the ERP analyses, ADOS CSS scores were examined continuously. Therefore, these plots do not directly reflect the analyses, but illustrate some of the effects observed in the continuous data.

There was also a significant interaction between participant group, stimulus familiarity, and Overall CSS on N290 amplitude, *F*_(2, 4680)_ = 10.26, *p* < 0.001, $n_{p}^{2}$ = 0.004, 95% CI [0.00, 0.01]. We examined responses to familiar and novel stimuli separately. In response to familiar stimuli, there was a significant interaction of Overall CSS and group, *F*_(2,2334)_ = 25.43, *p* < 0.001, $n_{p}^{2}$ = 0.02, 95% CI [0.01, 0.03]. As with responses to faces, increased (more negative) N290 amplitude to familiar stimuli were associated with higher Overall CSS for infants with FXS, *F*_(1, 658)_ = 9.72, *p* = 0.002, $n_{p}^{2}$ = 0.02, 95% CI [0.00, 0.04], and ASIBs, *F*_(1, 838)_ = 24.47, *p* < 0.001, $n_{p}^{2}$ = 0.03, 95% CI [0.01, 0.05], while decreased (more positive) N290 amplitude was associated with higher Overall CSS for LRC infants, *F*_(1, 838)_ = 26.07, *p* < 0.001, $n_{p}^{2}$ = 0.03, 95% CI [0.01, 0.06]. There was also a significant interaction of group and Overall CSS in response to novel stimuli, *F*_(2,2346)_ = 4.33, *p* = 0.013, $n_{p}^{2}$ = 0.004, 95% CI [0.00, 0.01]. Similar to N290 responses to toys, more negative N290 responses to novel stimuli were associated with higher Overall CSS for ASIBs, *F*_(1, 838)_ = 5.79, *p* = 0.0163, $n_{p}^{2}$ = 0.01, 95% CI [0.00, 0.02]. N290 responses to novel stimuli were not associated with Overall CSS for infants with FXS, *p* = 0.10, $n_{p}^{2}$ = 0.004, 95% CI [0.00, 0.02], and LRC infants, *p* = 0.416, $n_{p}^{2}$ = 0.001, 95% CI [0.00, 0.01].

#### Social Affect CSS

There was a significant interaction between participant group, stimulus type, and SA CSS on N290 amplitude, *F*_(2, 1188)_ = 4.21, *p* = 0.015, $n_{p}^{2}$ = 0.01, 95% CI [0.00, 0.02]. To better understand this interaction, we examined responses to faces and toys separately. There was a significant interaction of SA CSS and group for N290 responses to faces, *F*_(2, 594)_ = 16.72, *p* < 0.001, $n_{p}^{2}$ = 0.05, 95% CI [0.02, 0.09]. For ASIBs, a more negative N290 in response to faces was associated with higher SA CSS, *F*_(1, 214)_ = 17.53, *p* < 0.001, $n_{p}^{2}$ = 0.08, 95% CI [0.02, 0.15]. Once again, the opposite pattern was seen for LRC infants, who showed that more positive N290 amplitude in response to faces was related to higher SA CSS, *F*_(1, 214)_ = 24.49, *p* < 0.001, $n_{p}^{2}$ = 0.10, 95% CI [0.04, 0.18]**. **For infants with FXS, there was no relationship between N290 responses to faces and SA CSS, *p* = 0.384, $n_{p}^{2}$ = 0.005, 95% CI [0.00, 0.05]. There was also a significant interaction of SA CSS and group for N290 responses to toys, *F*_(2, 594)_ = 11.35, *p* < 0.001, $n_{p}^{2}$ = 0.04, 95% CI [0.01, 0.07]. More negative N290 responses to toys were associated with higher SA CSS for ASIBs, *F*_(1, 214)_ = 20.24, *p* < 0.001, $n_{p}^{2}$ = 0.09, 95% CI [0.03, 0.16], but the opposite pattern was seen for infants with FXS, *F*_(1, 166)_ = 4.13, *p* = 0.044, $n_{p}^{2}$ = 0.02, 95% CI [0.00, 0.09]. N290 responses to toys were not significantly associated with SA CSS for LRC infants, *p* = 0.088, $n_{p}^{2}$ = 0.01, 95% CI [0.00, 0.06].

There was also a significant interaction between participant group, stimulus familiarity, and SA CSS on N290 amplitude, *F*_(2, 4680)_ = 13.45, *p* < 0.001, $n_{p}^{2}$ = 0.01, 95% CI [0.00, 0.01]. We examined responses to familiar and novel stimuli separately. There was a significant interaction of SA CSS and group for N290 responses to familiar stimuli, *F*_(2, 2334)_ = 40.03, *p* < 0.001, $n_{p}^{2}$ = 0.03, 95% CI [0.02, 0.05]. A more negative N290 to familiar stimuli was associated with higher SA CSS for infants with FXS, *F*_(1, 658)_ = 4.64, *p* = 0.032, $n_{p}^{2}$ = 0.01, 95% CI [0.00, 0.03], and ASIBs, *F*_(1,838)_ = 53.96, *p* < 0.001, $n_{p}^{2}$ = 0.06, 95% CI [0.03, 0.09]. More positive N290 amplitude in response to familiar stimuli for LRC infants was related to higher SA CSS, *F*_(1, 838)_ = 34.51, *p* < 0.001, $n_{p}^{2}$ = 0.04, 95% CI [0.02, 0.07]. There was a significant interaction of SA CSS and group in response to novel stimuli, *F*_(2, 2346)_ = 6.66, *p* = 0.001, $n_{p}^{2}$ = 0.01, 95% CI [0.00, 0.01]. More positive N290 responses to novel stimuli were associated with higher SA CSS for infants with FXS, *F*_(1,658)_ = 12.78, *p* < 0.001, $n_{p}^{2}$ = 0.02, 95% CI [0.00, 0.05]. N290 responses to novel stimuli were not associated with SA CSS for ASIBs, *p* = 0.225, $n_{p}^{2}$ = 0.002, 95% CI [0.00, 0.01], and LRC infants, *p* = 0.065, $n_{p}^{2}$ = 0.004, 95% CI [0.00, 0.02].

#### Restricted and Repetitive Behaviors CSS

There was a significant interaction between participant group, stimulus type, and RRB CSS on N290 amplitude, *F*_(2, 1188)_ = 9.30, *p* < 0.001, $n_{p}^{2}$ = 0.02, 95% CI [0.00, 0.03]. To better understand this interaction, we examined responses to faces and toys separately. The interaction of RRB CSS and group in response to faces was not significant, *p* > 0.05, $n_{p}^{2}$ = 0.002, 95% CI [0.00, 0.01]. However, there was a significant interaction of RRB CSS and group in response to toys, *F*_(2,594)_ = 16.38, *p* < 0.001, $n_{p}^{2}$ = 0.05, 95% CI [0.02, 0.09]. More negative N290 responses to toys were associated with higher RRB CSS for ASIBs, *F*_(1, 214)_ = 28.89, *p* < 0.001, $n_{p}^{2}$ = 0.12, 95% CI [0.05, 0.20], but the opposite pattern was shown by infants with FXS, *F*_(1,166)_ = 12.93, *p* < 0.001, $n_{p}^{2}$ = 0.07, 95% CI [0.02, 0.16]. N290 responses to toys were not associated with RRB CSS for LRC infants, *p* = 0.144, $n_{p}^{2}$ = 0.01, 95% CI [0.00, 0.05].

There was also a significant interaction between participant group, stimulus familiarity, and RRB CSS on N290 amplitude, *F*_(2, 4680)_ = 9.27, *p* < 0.001, $n_{p}^{2}$ = 0.003, 95% CI [0.00, 0.01]. We examined responses to familiar and novel stimuli separately. The interaction of RRB CSS and group in response to familiar stimuli was not significant, *F*_(2, 2334)_ = 1.82, *p* = 0.163, $n_{p}^{2}$ = 0.002, 95% CI [0.00, 0.01]. There was a significant interaction of RRB CSS and group in response to novel stimuli, *F*_(2, 2346)_ = 14.62, *p* < 0.001, $n_{p}^{2}$ = 0.01, 95% CI [0.01, 0.02]. More negative N290 responses to novel stimuli were associated with higher RRB CSS for ASIBs, *F*_(1, 838)_ = 13.15, *p* = 0.003, $n_{p}^{2}$ = 0.02, 95% CI [0.00, 0.04], and LRC infants, *F*_(1,850)_ = 13.76, *p* < 0.001, $n_{p}^{2}$ = 0.02, 95% CI [0.00, 0.04]. The opposite pattern of responses was seen for infants with FXS, *F*_(1,658)_ = 8.85, *p* = 0.003, $n_{p}^{2}$ = 0.01, 95% CI [0.00, 0.04].

### P400

**Figure 3:**
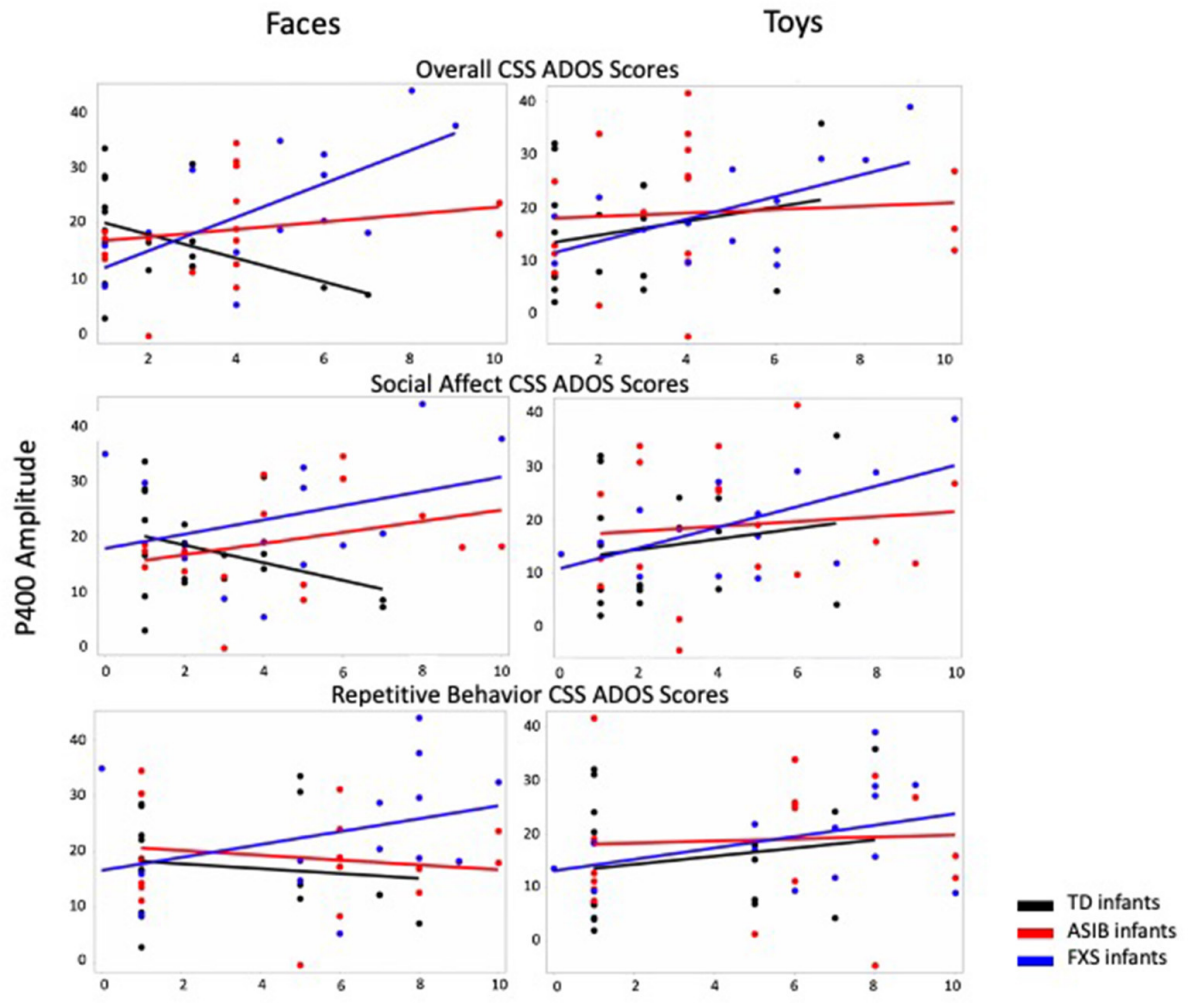
P400 amplitude to faces and toys across ASIBs, infants with FXS, and LRC infants is presented in relation to Overall, Social Affect, and Restricted and Repetitive Behavior calibrated severity scores. Average P400 amplitude is calculated across Parietal Occipital, Occipital, and Inion electrodes.

Graphs presenting the relations between P400 amplitude to faces and toys across ASIBs, infants with FXS, and LRC infants and Overall, Social Affect, and Restricted and Repetitive Behavior CSS are presented in Figure 3.

#### Overall CSS

There was a significant interaction between participant group, stimulus type, and Overall CSS on P400 amplitude, *F*_(2, 988)_ = 4.32, *p* = 0.014, $n_{p}^{2}$ = 0.01, 95% CI [0.00, 0.02]. There was a significant interaction of Overall CSS and group in response to faces, *F*_(2, 494)_ = 30.66, *p* < 0.001, $n_{p}^{2}$ = 0.11, 95% CI [0.06, 0.16]. For infants with FXS, a more positive amplitude P400 response to faces was associated with higher Overall CSS, *F*_(1,138)_ = 61.39, *p* < 0.001, $n_{p}^{2}$ = 0.31, 95% CI [0.19, 0.42]. However, for LRC infants, decreased (less positive) P400 amplitude in response to faces was related to higher Overall CSS, *F*_(1, 178)_ = 4.80, *p* = 0.030, $n_{p}^{2}$ = 0.03, 95% CI [0.00, 0.09]. There was no relation between P400 amplitude in response to faces and Overall CSS for ASIBs, *p* = 0.082, $n_{p}^{2}$ = 0.02, 95% CI [0.00, 0.07]. There was a significant interaction of Overall CSS and group in response to toys, *F*_(2, 494)_ = 11.86, *p* < 0.001, $n_{p}^{2}$ = 0.05, 95% CI [0.02, 0.08]. For ASIBs decreased (less positive) P400 amplitude in response to toys was related to higher Overall CSS, *F*_(1, 178)_ = 9.80, *p* = 0.002, $n_{p}^{2}$ = 0.05, 95% CI [0.01, 0.13]. Once again, for infants with FXS, a more positive P400 in response to toys was associated with higher Overall CSS, *F*_(1, 178)_ = 15.74, *p* < 0.001, $n_{p}^{2}$ = 0.10, 95% CI [0.03, 0.20]. There was no relation between P400 amplitude in response to toys and Overall CSS for LRC infants, *p* = 0.388, $n_{p}^{2}$ = 0.004, 95% CI [0.00, 0.04]. These effects are observed in the ERP plots presented in Figure 2. The P400 is the positive peak occurring at approximately 400–500 ms after stimulus onset. Participants with FXS with high Overall CSS demonstrated a greater amplitude P400 response than those with low Overall CSS. Alternatively, ASIBs with high Overall CSS show a decreased P400 amplitude in response to toys compared to ASIBs with low Overall CSS.

There was also a significant interaction between participant group, stimulus familiarity, and Overall CSS on P400 amplitude, *F*_(2, 3898)_ = 14.54, *p* < 0.001, $n_{p}^{2}$ = 0.01, 95% CI [0.00, 0.01]. We examined responses to familiar and novel stimuli separately. There was a significant interaction of Overall CSS and group in response to familiar stimuli, *F*_(2, 1944)_ = 8.10, *p* < 0.001, $n_{p}^{2}$ = 0.01, 95% CI [0.00, 0.02]. Infants with FXS showed a more positive P400 associated with higher Overall CSS, *F*_(1, 548)_ = 6.41, *p* = 0.012, $n_{p}^{2}$ = 0.01, 95% CI [0.00, 0.04], while ASIBs showed a less positive P400 in response to familiar stimuli was associated with higher Overall CSS, *F*_(1, 698)_ = 12.11, *p* < 0.001, $n_{p}^{2}$ = 0.02, 95% CI [0.00, 0.04]. There was no relation between P400 amplitude in response to faces and Overall CSS for LRC infants, *p* = 0.790, $n_{p}^{2}$ = 0.0001, 95% CI [0.00, 0.01]. There was a significant interaction of Overall CSS and group in response to novel stimuli, *F*_(2, 1954)_ = 48.19, *p* < 0.001, $n_{p}^{2}$ = 0.05, 95% CI [0.03, 0.07]. Infants with FXS showed a more positive P400 associated with higher Overall CSS, *F*_(1, 548)_ = 79.40, *p* < 0.001, $n_{p}^{2}$ = 0.13, 95% CI [0.08, 0.18], but ASIBs showed a less positive P400 in response to novel stimuli was associated with higher Overall CSS, *F*_(1, 698)_ = 6.34, *p* = 0.012, $n_{p}^{2}$ = 0.01, 95% CI [0.00, 0.03]. There was no relation between P400 amplitude in response to faces and Overall CSS for LRC infants, *p* = 0.078, $n_{p}^{2}$ = 0.004, 95% CI [0.00, 0.02].

#### Social Affect CSS

The interaction between participant group, stimulus type, and SA CSS was not significant, *p* = 0.460, $n_{p}^{2}$ = 0.002, 95% CI [0.00, 0.01]. However, there was a significant interaction between participant group and SA CSS on P400 amplitude, *F*_(2, 988)_ = 4.00, *p* = 0.019, $n_{p}^{2}$ = 0.01, 95% CI [0.00, 0.02]. For infants with FXS, a higher SA CSS was significantly associated with higher P400 amplitude, *F*_(1, 138)_ = 42.09, *p* < 0.001, $n_{p}^{2}$ = 0.23. P400 amplitude was not significantly associated with SA CSS for ASIBs, *p* = 0.083, $n_{p}^{2}$ = 0.02, or LRC infants, *p* = 0.853, $n_{p}^{2}$ = 0.0002.

There was a significant interaction between participant group, stimulus familiarity, and SA CSS on P400 amplitude, *F*_(2, 3898)_ = 14.72, *p* < 0.001, $n_{p}^{2}$ = 0.01, 95% CI [0.00, 0.01]. There was a significant interaction of SA CSS and group in response to familiar stimuli, *F*_(2, 1944)_ = 10.28, *p* < 0.001, $n_{p}^{2}$ = 0.01, 95% CI [0.00, 0.02]. Infants with FXS showed a more positive P400 associated with higher SA CSS, *F* (1, 548) = 5.07, *p* =0.025, $n_{p}^{2}$ =0.01, 95% CI [.00,0.03], while ASIBs showed a less positive P400 in response to familiar stimuli was associated with higher SA CSS, *F*_(1, 698)_ = 13.57, *p* < 0.001, $n_{p}^{2}$ = 0.02, 95% CI [0.00, 0.04]. There was no relation between P400 amplitude in response to familiar stimuli and SA CSS for LRC infants, *p* = 0.067, $n_{p}^{2}$ = 0.01, 95% CI [0.00, 0.02]. There was a significant interaction of SA CSS and group in response to novel stimuli, *F*_(2, 1954)_ = 35.41, *p* < 0.001, $n_{p}^{2}$ = 0.04, 95% CI [0.02, 0.05]. Infants with FXS showed a more positive P400 associated with higher SA CSS, *F*_(1, 548)_ = 88.91, *p* < 0.001, $n_{p}^{2}$ = 0.14, 95% CI [0.09, 0.19]. There was no relation between P400 amplitude in response to novel stimuli and SA CSS for ASIBs, *p* = 0.862, $n_{p}^{2}$ = 0.00004, 95% CI [0.00, 0.01], and LRC infants, *p* = 0.401, $n_{p}^{2}$ = 0.001, 95% CI [0.00, 0.01].

#### Restricted and Repetitive Behavior CSS

The interaction between participant group, stimulus type, and RRB CSS on P400 amplitude was not significant, *F*_(2, 3898)_ = 1.45, *p* = 0.235, $n_{p}^{2}$ = 0.01, 95% CI [0.00, 0.02]. However, there was a significant interaction between participant group, stimulus familiarity, and RRB CSS on P400 amplitude, *F*_(2, 3898)_ = 33.90, *p* < 0.001, $n_{p}^{2}$ = 0.02, 95% CI [0.01, 0.03]. There was a significant interaction of RRB CSS and group in response to familiar stimuli, *F*_(2, 1944)_ = 6.25, *p* = 0.002, $n_{p}^{2}$ = 0.01, 95% CI [0.00, 0.02]. A less positive P400 in response to familiar stimuli was associated with higher RRB CSS for infants with FXS, *F*_(1, 548)_ = 27.47, *p* < 0.001, $n_{p}^{2}$ = 0.05, 95% CI [0.02, 0.09], and LRC infants**, ***F*_(1, 698)_ = 15.21, *p* < 0.001, $n_{p}^{2}$ = 0.02, 95% CI [0.01, 0.05]. There was no relation between P400 amplitude in response to familiar stimuli and RRB CSS for ASIBs, *p* = 0.200, $n_{p}^{2}$ = 0.002, 95% CI [0.00, 0.02]. There was a significant interaction of RRB CSS and group in response to novel stimuli, *F*_(2, 1954)_ = 33.09, *p* < 0.001, $n_{p}^{2}$ = 0.03, 95% CI [0.02, 0.05]. Infants with FXS showed a more positive P400 associated with higher RRB CSS, *F*_(1,548)_ = 18.79, *p* < 0.001, $n_{p}^{2}$ = 0.03, 95% CI [0.01, 0.07]. Less positive P400 amplitudes were associated with higher RRB CSS in ASIBs, *F*_(1, 698)_ = 41.16, *p* < 0.001, $n_{p}^{2}$ = 0.06, 95% CI [0.03, 0.09], and LRC infants, *F*_(1, 708)_ = 14.93, *p* < 0.001, $n_{p}^{2}$ = 0.02, 95% CI [0.01, 0.05].

### Nc

#### Overall CSS

There were no significant interactions between group, stimulus type, and Overall CSS on Nc amplitude, *F*_(1, 1161)_ = 0.09, *p* = 0.914. There were also no significant interactions between group, stimulus familiarity, and Overall CSS on Nc amplitude, *F*_(1, 1161)_ = 0.01, *p* = 0.991.

#### Social Affect CSS

There were no significant interactions between group, stimulus type, and SA CSS on Nc amplitude, *F*_(1, 1161)_ = 0.02, *p* = 0.979. Additionally, there were no significant interactions between group, stimulus familiarity, and SA CSS on Nc amplitude, *F*_(1, 1161)_ = 1.73, *p* = 0.178.

#### Restricted and Repetitive Behavior CSS

There were no significant interactions between group, stimulus type, and RRB CSS on Nc amplitude, *F*_(1, 1161)_ = 0.78, *p* = 0.460. There were also no significant interactions between group, stimulus familiarity, and RRB CSS on Nc amplitude, *F*_(1, 1161)_ = 2.48, *p* = 0.085.

## Discussion

Results of the current study establish relations between face processing ERP responses at 12 months of age and ADOS-2 calibrated severity scores in early childhood in FXS as well as siblings of children with ASD and low-risk controls. This work indicates that ERP responses in infancy may be predictive of later behavioral symptoms of ASD in infants at risk for ASD, whether it be syndromic risk or familial risk, and supports the use of ERPs as a measure to identify potential markers of ASD. However, the relations observed were complex and varied across group based on ERP component examined and stimulus category. This likely reflects the high level of heterogeneity in developmental trajectories and symptom profiles associated with ASD, which we were unable to investigate further due to our small sample size.

Results revealed that N290 amplitude was associated with ASD symptoms for all participant groups examined. Infants with FXS showed that greater amplitude (more negative) N290 responses to faces and familiar stimuli were associated with more severe ASD symptoms overall, while less negative N290 responses to toys and novel stimuli were associated with more severe social affect and restricted and repetitive behavior symptoms. ASIBs showed a consistent association between more negative N290 amplitude and greater severity of ASD symptoms. For ASIBs, more negative N290 amplitude to all stimuli were associated with more severe overall and social affect symptoms, while only more negative N290 amplitude responses to toys and novel stimuli were associated with more severe restricted and repetitive behavior symptoms. The LRC group showed an opposite pattern of responses, such that less negative N290 responses to faces and familiar stimuli were associated with more severe overall and social affect symptoms. The N170 has been implicated as a marker of ASD in children and adults and was recently submitted to the FDA's Center for Drug Evaluation and Research Biomarker Qualification Program with the intent to use N170 responses to better understand heterogeneity in ASD (61). The current work indicates that the N290 may hold promise in distinguishing amongst diverse groups of high-risk infants, as well.

Previous research has indicated that infants with FXS show enhanced N290 ERP activity in response to faces relative to ASIB and LRC groups, while ASIBs showed more muted ERP responses (13). It was hypothesized that these ERP effects may reflect a general hyperactive response to faces in infants with FXS vs. a hypoactive response in ASIBs. Therefore, it was surprising to find that for the both FXS and ASIB participant groups, more negative N290 responses were associated with more severe behavioral symptoms of ASD. This pattern of results was more robust for N290 responses to faces than to toys and to familiar than novel stimuli. When examining relations between N290 responses to toys and novel stimuli and ASD symptom severity, ASIBs continued to show that increased amplitude responses to toys and novel stimuli were associated with more severe symptoms. These results indicate that N290 responses to faces, especially familiar faces, may be informative when investigating ASD risk across discrete etiologies. Furthermore, across all stimulus types, the current results suggest that enhanced responses at the neural level in infancy are more predictive of later developing ASD symptoms than more muted responses.

The P400 amplitude at 12 months of age was also associated with the later development of symptoms of ASD. Greater amplitude P400 responses to all stimuli were associated with more severe overall and social affect symptoms among children with FXS. Decreased P400 amplitude (to familiar stimuli, novel stimuli, and to toys) were associated with higher symptom severity in ASIBs. Typically-developing LRCs showed the opposite effect for P400 responses to faces, where less positive P400 amplitude was linked to more severe ASD symptoms overall. ASIBs showed no significant relations between P400 amplitude to faces and ASD symptom severity. It was surprising that there were strong associations between P400 amplitude and ASD symptom severity in high risk infants, as no significant effects of P400 were observed across participant groups in infancy (13). However, significant effects of participant group and ASD symptom severity on Nc amplitude were not observed.

Relations between the N290 and P400 and later ASD symptoms are consistent with other studies examining links between infant social information processing and ASD outcomes (33). Both the N290 and P400 are believed to reflect infant face processing and enhanced or muted ERP responses may be reflective of atypical social processing. Reduced or atypical patterns of attention to faces have been reported in several behavioral studies of infants later diagnosed with ASD [e.g., (17, 21, 61, 62)]. Reduced engagement during social information processing may disrupt social development, eventually contributing to the development of symptoms of ASD. Gui and colleagues (63) recently reported decreased differentiation of face and object stimuli at the N290 among infants later diagnosed with ASD. Additionally, Shephard and colleagues (64) found that ASIBs with more negative N290 amplitude responses to faces relative to noise scored higher in social communication problems at 7 years of age. Their results indicated that responses to noise stimuli were largely responsible for this effect (i.e., more positive N290 responses were associated with higher social communication problems). Studies by Elsabbagh and colleagues (48) and Buss and colleagues (65) have found that greater P400 amplitude to faces with direct gaze compared with averted gaze was associated with ASD symptoms at 3 years of age. Overall, these findings support our results suggesting that face-sensitive ERPs in infancy may provide insight into later ASD symptoms.

It was surprising that only the face-sensitive N290 and P400 were associated with ASD symptoms, and not the Nc ERP component. The Nc was differentiated across all three groups in infancy (13), and we had expected that unqiue patterns of Nc responses may be associated with unique symptom profiles. The Nc is associated with engagement of attention, and differences in Nc amplitude have been reported between infants at high risk for ASD and control participants during infancy [e.g., (28, 30, 65, 66)]. Although Nc amplitude has been associated with infant risk status, its relation to ASD outcomes is less clear. Our results are consistent with one recent study finding no connection between mean Nc amplitude at 8 months of age and later ASD (67). Another study reported differences in Nc amplitude between 6-month-old infants later diagnosed with ASD and control participants, but not 12-month-olds later diagnosed with ASD and control participants (68). We had expected that Nc amplitude would be associated with ASD symptoms in our sample of FXS, ASIBs, and LRC infants, due to unique patterns of Nc activation observed across these groups (13), but all of the current Nc analyses were nonsignificant. Relations between Nc amplitude and ASD outcomes should be further investigated in future research, due to sparse and conflicting results.

Additional work in this area is needed to better understand how neural responses differ across infants with FXS based on ASD diagnosis and how infants with FXS that receive an ASD diagnosis uniquely process stimuli from ASIBs that receive an ASD diagnosis. Small sample size was a significant limitation in the current study. While current sample sizes restricted us from analyzing outcomes categorically, we aim to expand our sample to better understand how infant ERP responses across these groups are associated with diagnostic outcomes in future research. Further work in this area with larger samples will allow us to examine the role of high-risk subgroups (e.g., sex, cognitive function) in infant ERP responses and ASD outcomes. There were more females (i.e., 50%) in the sample of children with FXS in the current study than is typical. This may have impacted our results, as a smaller subset was diagnosed was diagnosed with ASD than has been reported in larger scale studies of ASD in preschool children with FXS (i.e., 36 vs. 61%; 9). Additionally, the inclusion of multiple comparisons in our statistical approach may have allowed for Type I errors. Methods were adopted to help to prevent Type I errors and effect sizes, including 95% confidence intervals for effect sizes, were reported to further describe comparisons, however, replication of this work will be important to confirmation of the current findings. Additional work with a large sample of infants with FXS would allow for investigation of the role of gender in relations between infant ERP responses and later developing symptoms of ASD. Furthermore, FXS is associated with intellectual impairment, and it is important to examine how the presence of intellectual disability may impact stimulus processing in this sample. Adoption of additional control groups that are impacted by intellectual disability, but less likely to develop ASD, such as infants with Down syndrome, may allow for better understanding of how unique patterns of neural responses in infants with FXS are associated with intellectual disability. Another goal of this line of research is to expand the investigation to other high-risk participant groups. For example, Feldman and colleagues (69) found that infants in families with a greater number of medical conditions were more likely to demonstrate ASD symptoms. It would be valuable to investigate neural responses and ASD symptoms in this and other high-risk groups to better understand heterogeneous pathways to ASD.

Results of the current study highlight that neural correlates of face processing in infancy are associated with later behavioral symptoms of ASD in children with FXS. This work is highly valuable, especially as this was the first study to examine relations between infant ERPs and symptoms of ASD in a high-risk group beyond ASIBs. By including multiple groups of participants at high risk for ASD, we were able to examine heterogeneity in relations between infant neural responses and childhood ASD symptoms. Despite the observation of unique patterns of neural responses in infancy across infants with FXS and ASIBs, similar patterns of infant ERP responses were associated with childhood ASD symptom severity for both groups. Specifically, we found that greater N290 amplitude in response to faces in infancy was associated with the presentation of more severe symptoms of ASD in early childhood across both infants with FXS and ASIBs. However, less consistent results were observed across high-risk groups for the P400 and Nc components. It will be important to further investigate the utility of the N290, P400, and Nc ERP components as potential markers for ASD in future studies enrolling large and diverse high-risk samples. It is our intention that this work will eventually contribute to the ability to identify valid and reliable ERP markers evident at the level of the individual, promoting early intervention and treatment among infants and toddlers most likely to receive a diagnosis of ASD.

## Data Availability Statement

The clinical and demographic data for this study are available as part of the National Database for Autism Research (https://nda.nih.gov/edit_collection.html?id=1888). Contact the authors to access additional data.

## Ethics Statement

The studies involving human participants were reviewed and approved by University of South Carolina Institutional Review Board. Written informed consent to participate in this study was provided by the participants' legal guardian/next of kin.

## Author Contributions

MG, JRi, AH, and JRo contributed to the conception and design of the study. JRi and JRo led recruitment and data collection efforts. AH organized the clinical data and assisted in its use and interpretation. MG performed the statistical analyses, created the figures and table, and wrote the first draft of the manuscript. All authors contributed to manuscript revision and approved the submitted version.

## Funding

This research was supported by grants R37 HD018942 to JRi and NIMH-R01MH090194 to JRo.

## Conflict of Interest

The authors declare that the research was conducted in the absence of any commercial or financial relationships that could be construed as a potential conflict of interest.

## Publisher's Note

All claims expressed in this article are solely those of the authors and do not necessarily represent those of their affiliated organizations, or those of the publisher, the editors and the reviewers. Any product that may be evaluated in this article, or claim that may be made by its manufacturer, is not guaranteed or endorsed by the publisher.
